# Account of Diseases in an Eastern District of London

**Published:** 1802-10-01

**Authors:** 


### ACCOUNT OF DISEASES IN AN EASTERN DISTRICT OF LONDON,

*From August* 20, *to September* 20, 1802.

| | |
|---|---|
| ACUTE DISEASES. | |
| Typhus | 4 |
| Dyſenteria | 7 |
| Cholera | 46 |
| Rheumatiſmus Acutus | 3 |
| CHRONIC DISEASES. | |
| Tuſſis | 5 |
| Dyſpnœa | 4 |
| Tuſſis cum Dyſpnœa | 8 |
| Hæmoptyſis | 3 |
| Hydrothorax | 4 |
| Acites | 3 |
| Anaſarca | 4 |
| Cephalalgia | 5 |
| Paralyſis | 2 |
| Diarrhœa | 20 |
| Enterodynia | 5 |
| Hæmorrhois | 3 |
| Amenorrhœa | 10 |
| Fluor Albus | 7 |
| Menorrhagia | 5 |
| Dyſuria | 4 |
| Rheumatiſmus Chronicus | 7 |
| PUERPERAL DISEASES. | |
| Menorrhagia Lochialis | 5 |
| Dolores poſt Partum | 7 |
| Maſtodynia | 4 |
| Ragas Papillæ | 3 |
| INFANTILE DISEASES. | |
| Eryſipelas Infantilis | 3 |
| Pertuſſis | 2 |
| Ophthalmia | 6 |
| Ophthalmia Purulenta | 4 |

In the liſt of diſeaſes prevailing for ſome weeks, thoſe which have their ſeat in ſome part of the alimentary canal have formed the largeſt proportion. That which makes a principal figure in the liſt for the preſent month is the Cholera Morbus. Of patients labouring under this, the number has been uncommonly large. This diſeaſe, indeed, may be conſidered as the epidemic of the Autumnal Months. It is characterized by a very copious diſcharge from the ſtomach and bowels; Purging and vomiting are the moſt prominent ſymptoms. Theſe, in ſome caſes, go on at the ſame time; but, in others, they occur alternately. The fluid diſcharged is of a bilious nature. Dr. Saunders obſerves, "that it is bile in a very diſeaſed ſtate:" And he thinks it probable, "that from the quantity ſecreted, and the rapid manner in which it is poured into the duodenum, there is not time ſufficient for a perfect ſecretion, that the fluid therefore fore is somewhat of an intermediate nature between blood and bile."

The disease is often preceded by uneasiness in the stomach and bowels; a sense of fulness about the præcordia, heartburn, and flatulence sometimes give warning of its approach; and in the course of the disease there is often not only pain, but considerable distention of the abdomen. Spasmodic affections of the lower extremities also form a symptom of the complaint. Retention of urine is, in some instances, a source of additional distress to the patient; and if these symptoms are accompanied by general convulsions, a fatal termination must be expected. Though this disease seems to depend for its *principal* occasional cause on the heat which prevails at this season of the year, or on the sudden change of temperature; yet it may often be produced more hastily by filling the stomach with large quantities of indigestable food.

In the number of cases referred to in the list not any one proved fatal. The cure was in general conducted by dilution of the contents of the stomach and bowels, by some emollient and demulcent liquids; and by interposing an anodyne, as it was found necessary for the abatement of pain, or for the removal of spasm in the intestines, or any other parts of the body.

